# In-Situ Measurement of Gas Permeability for Membranes in Water Electrolysis

**DOI:** 10.3390/membranes15050147

**Published:** 2025-05-13

**Authors:** Shuaimin Li, Chuan Song, Li Xu, Yuxin Wang, Wen Zhang

**Affiliations:** 1 State Key Laboratory of Chemical Engineering and Low-Carbon Technology, School of Chemical Engineering and Technology, Tianjin University, Tianjin 300350, China; 2 Tianjin Key Laboratory of Membrane Science & Desalination Technology, School of Chemical Engineering and Technology, Tianjin University, Tianjin 300350, China; 3 National Industry-Education Integration Platform of Energy Storage, Tianjin University, Tianjin 300350, China

**Keywords:** gas barrier, H_2_ permeation, water electrolysis, in-situ measurement, electrolyte membranes, Zirfon, PPS, FAA-3-PK-75, BILP-PE, electrode configuration

## Abstract

Water electrolysis (WE) is a green technology for producing hydrogen gas without the emission of carbon dioxide. The ideal membrane materials in WE should be capable of transporting ions quickly and have gas barrier properties in harsh work environments. However, currently, no desirable measurement method has been developed for evaluating the gas barrier behavior of the membranes. Hence, an in-situ electrochemical method is developed to measure the gas permeability of membranes in the actual electrolysis environment, with the supersaturated state of H_2_ in the electrolyte and H_2_ bubbles during the electrolysis process. Four membranes, including Zirfon (a state-of-the-art alkaline WE membrane), polyphenylene sulfide fabric (PPS, a commercial alkaline WE membrane), FAA-3-PK-75 (a commercial anion-exchange membrane), and BILP-PE (a home-made composite membrane) were employed as the standard samples to perform the electrochemical measurement under different current densities, temperatures, and electrolyte concentrations. The results show that an increase in electrolytic current density or temperature or a decrease in KOH concentration can increase the H_2_ permeability of the membrane. The two porous membranes, Zirfon and PPS, are more affected by the current density and KOH concentration, while the dense FAA-3-PK-75 and BILP-PE membranes have a stronger ability to hinder H_2_ permeation. Under the conditions of 80 °C, 30 wt.% KOH, 101 kPa, and 400 mA·cm^−2^, the hydrogen permeability (×10^10^ L·cm·cm^−2^·s^−1^) of Zirfon, PPS, FAA, and BILP-PE are 263, 367, 28.3, and 5.32, respectively.

## 1. Introduction

Hydrogen is a very promising secondary energy carrier, with the advantages of high calorific value and no carbon emissions. Over the past years, water electrolysis, integrating renewable energy, has attracted increasing interest in producing green hydrogen [1]. However, the volatility of renewable energy places higher demands on thSSe materials of water electrolysis. The membranes are essential materials in water electrolysis technology, which play a key role in performance [2,3,4,5,6]. In water electrolysis, H_2_ generated at cathodes and O_2_ generated at anodes, can diffuse through the membrane, reducing the purity of the gas. The mixture of H_2_ and O_2_ also poses an explosion risk. Notably, the purity of H_2_ obtained from electrolysis is typically higher than that of O_2_, indicating that H_2_ permeability through the membrane exceeds that of O_2_. Consequently, measuring the H_2_ permeability of the membrane provides an objective method to evaluate the gas barrier performance.

Currently, the commonly used methods for testing the gas permeability of membranes include bubble point pressure [7,8], volume, concentration [9], and electrochemical methods [10,11,12,13,14,15]. The volume method and concentration method require additional devices, such as gas chromatography or a high-precision flowmeter, which increases the complexity and cost of the gas barrier test device. In contrast, the electrochemical method measures the limiting current of the hydrogen oxidation reaction (HOR) or oxygen reduction reaction (ORR) of H_2_ or O_2_ permeating the membrane at the detection electrode, respectively. For example, the O_2_ permeability of Nafion 212 membranes was determined by testing the ORR limiting current density [16]. At 80 °C and 100% RH, the O_2_ limiting current density was approximately 2.8 mA·cm⁻^2^, and the permeability was about 1.8 × 10⁻^13^ mol·cm·s⁻^1^·cm⁻^2^·kPa⁻^1^, with both values increasing with temperature and humidity. Similarly, Schalenbach et al. [17] measured the H_2_ permeability of a Zirfon diaphragm in an alkaline water electrolyzer using the HOR limiting current density, reporting a value of 1.5 × 10⁻^12^ mol·cm·s⁻^1^·bar⁻^1^ at 80 °C in 30 wt.% KOH. Electrochemical methods have become widely used for measuring membrane gas permeability due to their fast response, high precision, and ease of operation.

However, the current electrochemical method for measuring the gas barrier of membranes exhibits significant differences between test conditions and actual working environments, failing to accurately reflect membrane gas permeability in actual electrolytic conditions. In industrial water electrolysis, bubble nucleation requires extremely high levels of supersaturation [18]. This process creates a large concentration difference across the membrane, driving H_2_ permeation. Additionally, nanobubbles may also transmit through the membrane [19]. However, the current electrochemical method usually employs humidified gas phase H_2_ to flow through the membrane surface, and H_2_ is dissolved and permeates through the alkaline-impregnated membrane [16]. In this process, the H_2_ in the membrane is not supersaturated, which is quite different from the actual electrolysis environment. Therefore, new in-situ methods that better simulate industrial electrolytic conditions are necessary for measuring membrane gas permeability.

Hence, this study designs an in-situ method to measure the H_2_ permeability of membranes under a close-to-actual electrolysis environment. The in-situ method is compared with the commonly utilized electrochemical method. The H_2_ permeability of commercial membranes, including Zirfon UTP-500, polyphenylene sulfide (PPS) fabric, FAA-3-PK-75 (anion exchange membrane), and the homemade BILP-PE composite membrane [20], is systematically investigated under varying electrolysis current densities, temperatures, and alkali concentrations. This work aims to develop an in-situ method to quickly provide reliable gas transport and kinetic data across the membrane in water electrolysis.

## 2. Experimental Section

### 2.1. Experimental Material

Potassium hydroxide (KOH, 95%) and anhydrous ethanol (EtOH, AR) were purchased from Komiou Chemical Reagent Co., Ltd. (Tianjin, China). Sulfuric acid (H_2_SO_4_, 95–98%) and acetone (AR) were purchased from Lianlongbohua (Tianjin, China) Pharmaceutical Chemistry Co., Ltd. Nickel chloride hexahydrate (NiCl_2_·6H_2_O, 98%) was purchased from Bailingwei Technology Co., Ltd. (Beijing, China). Xanthan gum (food grade) was purchased from Meihua Amino Acid Co., Ltd. (Xinjiang, China). Deionized water (>1 MΩ·cm) was purchased from Yongqingyuan Distilled Water Business Department, Xiqing District, Tianjin, China.

Pt/C catalyst (HPT020, 20% Pt) was purchased from Hesen Electric Co. (Shanghai, China). Anionic polymer solution (PiperION A5 HCO3 5%) was from Versogen (DE, USA). PPS fabric (680 μm) was purchased from Toray Industries, Inc. (Tokyo, Japan). The Zirfon membrane (UTP-500) was purchased from Agfa (Mortsel, Belgium), the anion exchange membrane (FAA-3-PK-75) was purchased from Fumatech (Bietigheimer Schloss, Germany), and the BILP-PE composite membrane (M-0.4-24) was made according to our previous work [20]. Nickel mesh (200 mesh) and nickel foam (δ 0.3 cm, 99.98%) were purchased from Huirui Mesh Manufacturing Factory, Hebei, China. Nickel plates (δ 0.3 cm, 99.98%) were purchased from Jinggong Metal Materials Co. (Danyang, China).

### 2.2. Methods of Measurement

#### 2.2.1. Principle of Measurement

**Figure 1:**
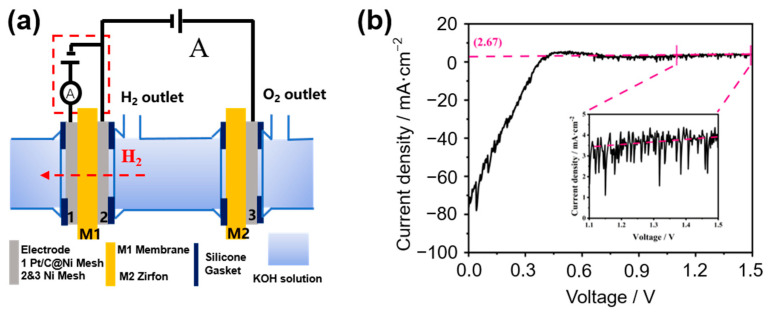
(**a**) Schematic diagram of the in-situ method for the H_2_ permeability of the membrane (M1); (**b**) Typical LSV curve for the in-situ method of the membrane’s HOR limiting current density.

The in-situ method for measuring the H_2_ permeability of the membrane is shown in Figure 1a. The negative pole of DC power supply A is connected to electrode 2 (nickel mesh, hydrogen-extraction reaction (HER) electrode) to electrolyze water to produce H_2_. The positive pole of DC power supply A is connected to electrode 3 (nickel mesh, oxygen-extraction reaction (OER) electrode) to electrolyze water to produce O_2_. The membrane to be measured, M1, is tightly clamped between electrode 1 (a Pt/C-sprayed porous nickel mesh, the HOR detection electrode) and electrode 2. The H_2_ generated at electrode 2 permeates through the membrane M1 and diffuses into electrode 1. Applying a voltage between electrodes 1 and 2 (electrode 1 is the anode), H_2_ undergoes a HOR reaction at electrode 1, generating a HOR current. At the beginning of the reaction, the reaction at both electrodes is controlled by the reaction kinetics, and the HOR current increases with the increase of voltage; ideally, when the diffusion of H_2_ controls the HOR, the current value does not increase anymore with the increase of voltage but stays constant. The value of the limiting current of the HOR corresponds to the permeability of H_2_ under the measurement conditions. The electrochemical workstation (CHI760E) measures the limiting current in the red dashed box in Figure 1a.

#### 2.2.2. Preparation of Electrodes

In Figure 1a, the glass electrolytic cell holds the KOH electrolyte to provide an electrolytic environment. The detection area of membranes is 2.27 cm^2^. Also, 200-mesh nickel meshes are used as the HER electrode (Electrode 2) and OER electrode (Electrode 3). The electrodes 2 and 3 function to form a simple electrolytic cell to generate hydrogen in situ, so we chose the simplest porous nickel mesh as the electrode. The HOR electrode and the HER electrode are both close to the membrane to be tested, and the OER electrode is also close to the Zirfon membrane. The distance between the HER electrode and the Zirfon membrane is 10 cm.

The HOR detection electrode is a porous nickel mesh loaded with Pt/C (Pt/C @ porous Ni mesh, Electrode 1), which was prepared as follows.

The nickel mesh and the nickel foam were ultrasonically cleaned in acetone and 1 M H_2_SO_4_ for 15 min and then rinsed with deionized water. Then, 7.11 g NiCl_2_·6H_2_O, 16.05 g NH_4_Cl, 0.06 g xanthan gum, and 150 mL of deionized water were put into a beaker, mixed, and ultrasonicated for 30 min to obtain the plating solution. The nickel mesh with an effective area of 3 cm × 3 cm was used as the cathode, and the nickel foam was used as the anode. The two electrodes were put in the above plating solution (160 mL) for 20 min at 0.2 A·cm^−2^. The electroplated nickel mesh was dried in a vacuum oven at 80 °C for 12 h to obtain porous nickel mesh.

For the catalyst ink, 0.02 g of Pt/C catalyst, 0.1 g of 5% PiperION solution, 0.5 mL of deionized water, and 3 mL of ethanol were put in a glass vial and treated by sonicating for 1 h. The porous nickel mesh was placed on a heating table at 60 °C, and the catalyst ink was sprayed onto the surface of the porous nickel mesh with a spray gun to obtain the HOR detection electrode. The actual loading rate of Pt for the prepared detection electrode was about 0.4 mg·cm^−2^.

#### 2.2.3. Electrochemical Detection

The test device was assembled as shown in Figure 1a; the glass electrolytic cell was filled with a certain concentration of KOH solution, the positive and negative poles of the DC power supply were connected to the OER electrode, and the HER electrode and the electrochemical workstation was connected to the HOR detection circuit (the detection circuit shown in the red dashed box in Figure 1a. Linear sweep voltammetry (LSV) was performed using an electrochemical workstation with a scanning interval of 0–1.5 V and a scan rate of 5 mV·s^−1^.

Figure 1b shows a typical LSV plot for the limiting current density of HOR by the in-situ method. After 0.8 V, the current density is almost constant, indicating that the HOR is controlled by H_2_ diffusion, and the current density at this stage is the limiting current density. Ideally, the current density should remain constant after a certain voltage. However, in Figure 1b, the current density increases slightly in the high-voltage region. Since there is a certain amount of electron current across the membrane, which increases linearly with the voltage applied to both ends of the cell, the limiting current should be the sum of the HOR limiting current (which is temperature-dependent and voltage-independent) and the electron current (which is proportional to the voltage). Therefore, the electron current must be removed to obtain an accurate HOR limit current. As shown by the dashed line in Figure 1b, a linear fitting is made between 1.1 and 1.5 V. The intercept of the fitting line with the vertical axis corresponds to the actual HOR limiting current density.

At 0 V, the hydrogen concentration at electrode 2 is greater than at electrode 1. At this time, the system forms a concentration cell, so a negative current is generated (shown in the Figure S1). When the electrochemical workstation is applied with an external voltage, the system forms an electrolytic cell, and the hydrogen at electrode 2 diffuses across the membrane to electrode 1 to produce an electrochemical reaction. Therefore, the current gradually changes from negative to positive as the voltage increases.

#### 2.2.4. Calculation of the H_2_ Permeability P_H_

When the H_2_ permeating across the membrane completely undergoes the HOR reaction, the H_2_ permeation flux of the membrane, *J_H_* (L·cm^−2^·s^−1^), can be expressed according to Faraday’s law:

(1) $$J_{H}=\frac{j_{l}\times V_{m}}{2F}$$

In Equation (1), *j_l_* (mA·cm^−2^) denotes the HOR limiting current density, V_m_ (22.4 L·mol^−1^) denotes the molar volume of the gas in the standard state, and F (96,485 A·s·mol^−1^) denotes the Faraday constant.

The membrane’s H_2_ permeability *P_H_* (L·cm·cm^−2^·s^−1^) can be as:

(2) $$P_{H}=J_{H}\times \delta_{m}$$

In Equation (2), *δ_m_* (cm) denotes the thickness of the membrane.

## 3. Results and Discussion

### 3.1. Measured Results

The LSV curves (1.1~1.5 V sections) of H_2_ permeability of the four membranes, Zirfon, PPS, FAA, and BILP-PE, measured by the in-situ method at different conditions, are shown in Figures S2–S5. The HOR limiting current densities *j_l_* of the membranes and the H_2_ permeation fluxes *J_H_* and H_2_ permeability *P_H_* calculated from Equations (1) and (2) are listed in Tables S1–S4.

### 3.2. Comparison of In-Situ and Ex-Situ Electrochemical Measurement

**Figure 2:**
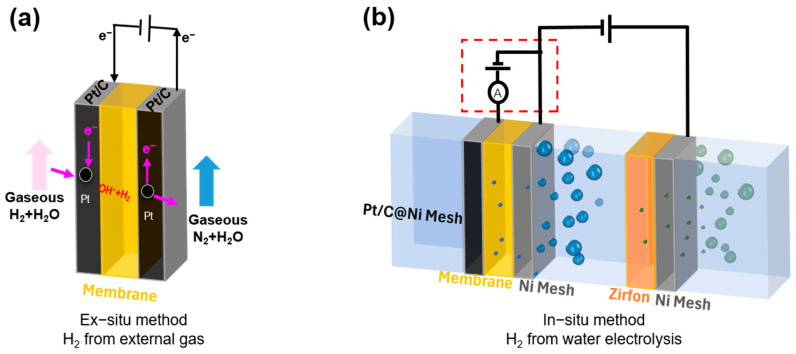
Schematic diagram of the (**a**) ex-situ (gas-phase) and (**b**) in-situ electrochemical method for the H_2_ permeability of the membrane.

In this part, we compared the in-situ and ex-situ electrochemical measurements. In the ex-situ method (Figure 2a) [20], the H_2_ passes through the electrode and diffuses into the membranes. In contrast, in the in-situ method test, the H_2_ generated on the electrode dissolves in the alkali solution, and the bubbles can be observed directly (Figure 2b). As we know, it can only nucleate and turn into bubbles when the H_2_ in the alkali solution reaches supersaturation several hundred times. That is, the H_2_ concentration difference on both sides of the membrane is much greater than that of the ex-situ method.

**Figure 3:**
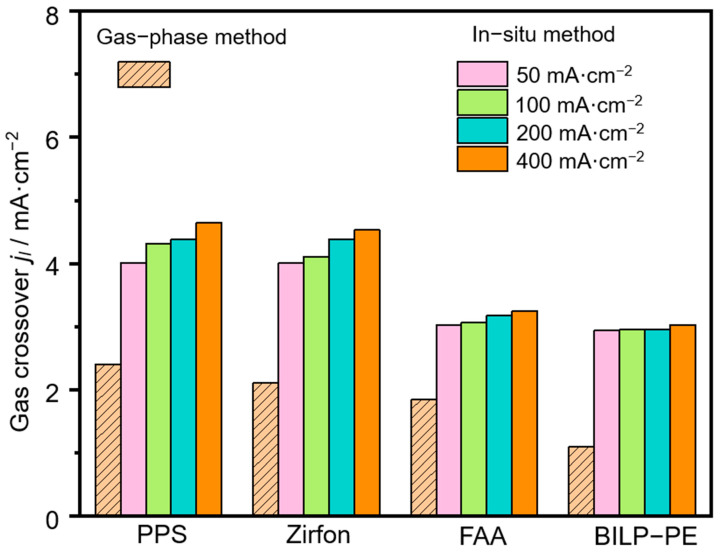
The HOR limiting current density of Zirfon, PPS, FAA, and BILP-PE was measured by in-situ and ex-situ methods. The in-situ method’s data were obtained in 30 wt.% KOH at 80 °C.

The data of the HOR limiting current density of Zirfon, PPS, FAA, and BILP-PE measured by the in-situ and ex-situ methods are reported in Figure 3 and Table 1. The results show that the limiting current density of HOR measured by the in-situ method is greater than that measured by the ex-situ method. In the ex-situ method, H_2_ on the cathode side dissolves in the membrane and diffuses to the HOR detection electrode. The solubility of H_2_ in the membrane is the saturated solubility of H_2_ under the test conditions. Compared with the ex-situ method, the H_2_ permeation rate in the membrane of the in-situ method is faster. Besides, some nanobubbles may pass through the membrane during the in-situ measurement. Therefore, the HOR limiting current density through the membrane in the in-situ method test is greater than that measured by the ex-situ method. We compared the in-situ method and other previous methods in Table S5. We used the uniform unit (×10^14^ mol·cm·s^−1^·cm^−2^·kPa^−1^) for the comparison. The results show that the H2 permeability measured by the in-situ method is greater than that measured by the ex-situ method (gas-phase method).

### 3.3. Effect of Electrolytic Current Density

**Figure 4:**
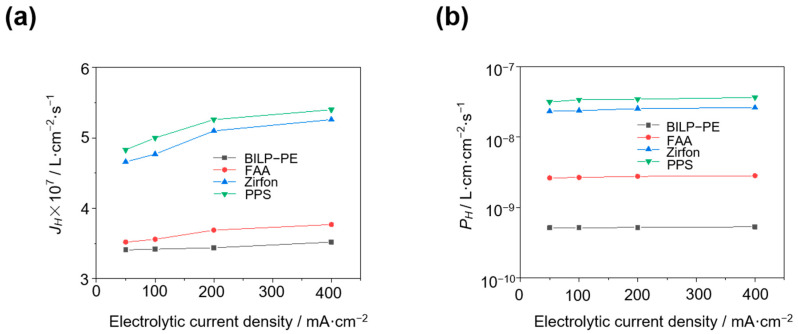
H_2_ permeation flux (**a**) and H_2_ permeability (**b**) of PPS, Zirfon, FAA, and BILP-PE at different electrolytic current densities in 30 wt.% KOH at 80 °C by the in-situ method.

The in-situ method measured the H_2_ permeation flux and H_2_ permeability of PPS, Zirfon, FAA, and BILP-PE at different electrolytic current densities in 80 °C and 30 wt.% KOH, as shown in Figure 4. The data comparison shows that the H_2_ permeability of the membrane increases slightly with the increase of electrolytic current density. Taking BILP-PE membrane as an example, the HOR limiting current density of BILP-PE composite membrane at 50, 100, 200, and 400 mA·cm^−2^ electrolytic current density is 2.94, 2.95, 2.96, and 3.03 mA·cm^−2^, respectively. The corresponding H_2_ permeability is 5.15 × 10^−10^, 5.16 × 10^−10^, 5.19 × 10^−10^, and 5.32 × 10^−10^ L·cm·cm^−2^·s^−1^, respectively. The results show that the H_2_ permeability of the BILP-PE composite membrane increases slightly with the increase of electrolytic current density. On the one hand, the increase in electrolysis current density increases the supersaturation of H_2_ in the solution [21], increasing the H_2_ concentration difference on both sides of the membrane, which promotes the permeation of H_2_ in the membrane. On the other hand, the increase in current density may reduce the volume of H_2_ bubbles [19], making it easier for bubbles to pass through the porous membrane, increasing the H_2_ permeability of the membrane.

Under the test conditions of 80 °C and 30 wt.% KOH, the electrolysis current density increases from 50 mA·cm^−2^ to 400 mA·cm^−2^, and the *P_H_* of the four membranes Zirfon, PPS, FAA, and BILP-PE increased by 12.9%, 11.9%, 7.20%, and 3.30%, respectively. The results show that compared with BILP-PE and FAA, the two porous membranes, PPS and Zirfon, are more affected by the electrolysis current density, indicating that the dense membrane has a stronger ability to hinder gas permeation.

### 3.4. Effect of Temperature

**Figure 5:**
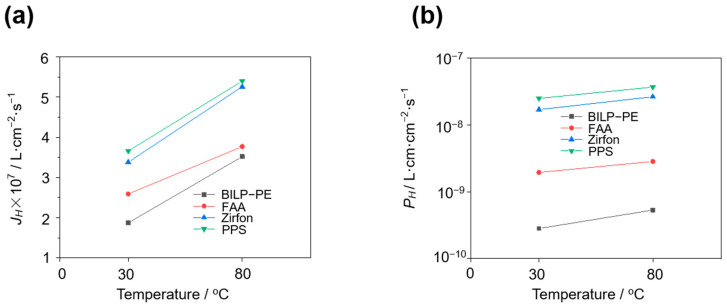
H_2_ permeation flux (**a**) and H_2_ permeability (**b**) of PPS, Zirfon, FAA, and BILP-PE at different temperatures in 30 wt.% KOH and the electrolytic current densities of 400 mA·cm^−2^ by the in-situ method.

At 30 wt.% KOH and 400 mA·cm^−2^, the H_2_ permeation flux and H_2_ permeability of four membranes, PPS, Zirfon, FAA, and BILP-PE, were tested at different temperatures by the in-situ method. In Figure 5, by comparing the data, it can be concluded that the H_2_ permeability of the membrane increases with increasing temperature. Taking the BILP-PE membrane as an example, under the test conditions of 30 wt.% KOH, the HOR limiting current density of the BILP-PE composite membrane at 30 °C and 80 °C was 1.61 and 3.03 mA·cm^−2^, respectively. As the temperature increases, the diffusion rate of H_2_ accelerates, and the H_2_ permeability of the membrane increases.

### 3.5. Effect of KOH Concentration

**Figure 6:**
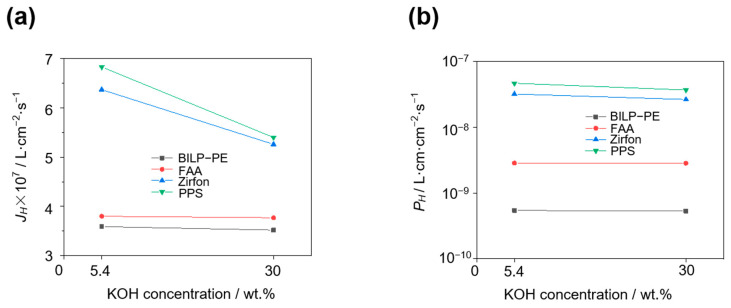
H_2_ permeation flux (**a**) and H_2_ permeability (**b**) of PPS, Zirfon, FAA, and BILP-PE at different KOH concentrations at 80 °C and the electrolytic current densities of 400 mA·cm^−2^ by the in-situ method.

At 80 °C and 400 mA·cm^−2^, the H_2_ permeation flux and H_2_ permeability of four membranes, PPS, Zirfon, FAA, and BILP-PE, at different KOH concentrations are shown in Figure 6. It can be found that the H_2_ permeability of the membrane decreases with the increase of KOH concentration. Taking the BILP-PE membrane as an example, under the conditions of 80 °C and 400 mA·cm^−2^, the HOR limiting current density of the BILP-PE composite membrane in 5.4 wt.% KOH and 30 wt.% KOH is 3.09 and 3.03 mA·cm^−2^, respectively. As the KOH concentration increases, the solubility of H_2_ in the KOH solution decreases [17,22], resulting in a decrease in the H_2_ permeability of the membrane.

Under the conditions of 80 °C and 400 mA·cm^−2^, when the KOH concentration increases from 5.4 wt.% to 30 wt.%, the *P_H_* of Zirfon, PPS, FAA, and BILP-PE membranes decreases by 17.6%, 20.9%, 0.702%, and 1.85%, respectively. The results show that compared with BILP-PE and FAA, the two porous membranes of PPS and Zirfon are more affected by the electrolyte concentration.

### 3.6. Position of HOR Electrode

**Figure 7:**
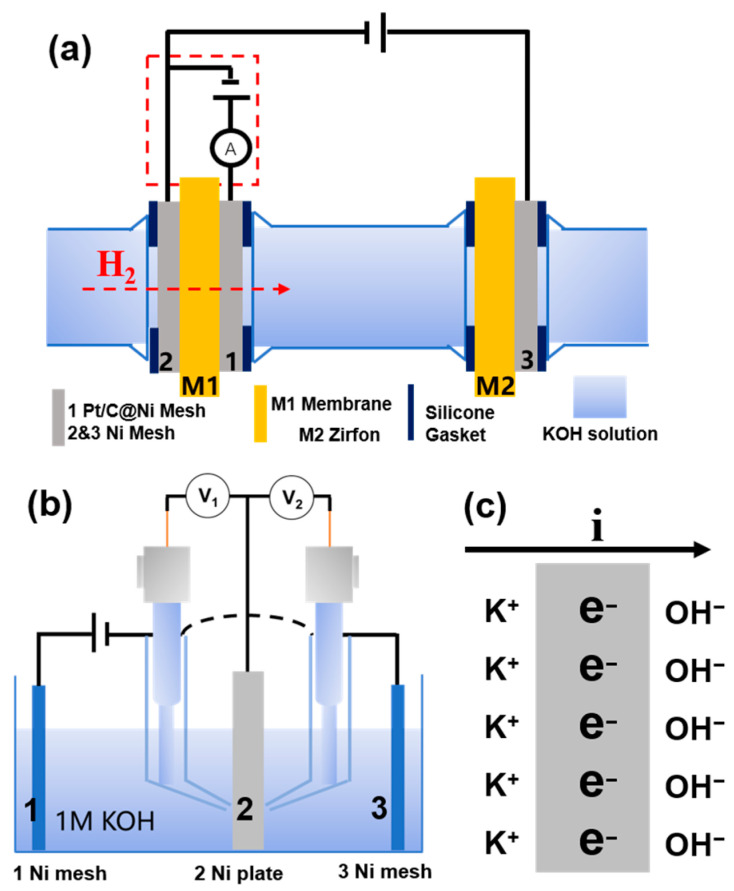
(**a**) The in-situ method with the HOR detection electrode between the anode and cathode of water electrolysis. (**b**) Schematic diagram of electrode polarization measurement. (**c**) Charge distribution of the polarized nickel plate.

In industrial water electrolysis, the membrane is located between the anode and cathode, and H_2_ may pass through the membrane from the cathode to the anode due to the electroosmotic drag. Therefore, when the HOR detection electrode is placed between the anode and cathode (as shown in Figure 7a), the measurement environment of the membrane is closer to the industrial reality. However, under these circumstances, the metal HOR electrode will be polarized in the electric field, and the polarized HOR detection electrode can become a bipolar plate. When the electrode potentials of HER and OER are reached, gas evolution reactions will occur on both sides of the electrode, and the oxidation current of H_2_ cannot be tested in this case.

To verify the polarization of the HOR electrode in Figure 7a, we placed two 3 cm × 3.5 cm nickel meshes opposite to each other, connected them to the positive and negative poles of the DC power, and put a 5 cm × 5 cm nickel plate between the two nickel meshes. The nickel mesh and plate were immersed in 1 M KOH (Figure 7b). At room temperature, the alkali solution was electrolyzed at different electrolysis current densities, and the potentials on the left side of the nickel plate (V1) and right side of the nickel plate (V2) were tested by the saturated calomel electrodes. When the electrolysis current density reached 40 mA·cm^−2^, V1 was −1.09 V, and V2 was 0.47 V. A small number of bubbles can be observed on the nickel plate. As the electrolysis current density increased, the potential on both sides of the nickel plate continued to increase, and the bubble generation became more intense (Table 2). This indicates that the nickel plate has been polarized. The reason for electrode polarization is that when the current passes through the nickel plate, the electrons in the nickel plate undergo directional migration (Figure 7c), resulting in a potential difference on both sides of the nickel plate.

Therefore, when testing the membrane’s H_2_ permeability, the HOR detection electrode must be placed outside the HER and OER electrodes to prevent electrode polarization.

## 4. Conclusions

This study designs an in-situ electrochemical method to test the H_2_ permeability of membranes in alkaline water electrolysis. It uses this method to explore the H_2_ permeability of Zirfon UTP-500, PPS fabric, FAA-3-PK-75, and BILP-PE membranes at different electrolysis current densities, temperatures, and KOH concentrations. The results of H_2_ permeability tested by this in-situ method and the commonly used ex-situ method were compared and analyzed. Compared with the ex-situ method, the in-situ method can simulate the in-situ hydrogen evolution environment and record the H_2_ permeation of membranes caused by the supersaturation of H_2_ in KOH solution and H_2_ bubbles. The increase in electrolysis current density increases the supersaturation of H_2_ in the solution, increasing the H_2_ concentration difference on both sides of the membrane, which promotes the permeation of H_2_ in the membrane. The increase in electrolysis current density may reduce the volume of H_2_ bubbles, promoting their permeation in the porous membrane and increasing its H_2_ permeability. As the temperature rises, the diffusion rate of H_2_ increases, and the membrane’s H_2_ permeability increases. As the KOH concentration increases, the solubility of H_2_ in the KOH solution decreases, reducing the membrane’s H_2_ permeability. Compared with the dense membranes of BILP-PE and FAA, the two porous membranes, PPS and Zirfon, are more affected by the electrolytic current density and the concentration of the electrolyte.

## Figures and Tables

**Table 1:** The H_2_ permeability of different membranes in the in-situ method (80 °C, 30% KOH, 101kPa, and 400 mA·cm^−2^).

Membranes	Thickness (μm)	IEC (meq·g^−1^)	*j_l_* (mA·cm^−2^)	*J_H_* × 10^7^ (L·cm^−2^·s^−1^)	*P_H_* × 10^10^ (L·cm·cm^−2^·s^−1^)
PPS Zirfon	500	—	4.65	5.40	367
	680	—	4.53	5.26	263
FAA BILP-PE	75.0	1.20–1.40	3.25	3.77	28.3
	15.1	—	3.03	3.52	5.32

**Table 2:** Potential on both sides of the nickel plate and phenomena on the nickel plate under different electrolytic current densities.

Electrolytic Current Densities/mA·cm^−2^	V_1_ vs. SCE/V	V_2_ vs. SCE/V	Phenomena on Nickel Plates
10	−0.10	0.23	No significant change
20	−0.56	0.32	No significant change
40	−1.09	0.47	Bubbles are produced
50	−1.11	0.55	A large number of bubbles are produced
60	−1.38	0.72	A large number of bubbles are produced

## Data Availability

The original contributions presented in this study are included in the article/Supplementary Materials. Further inquiries can be directed to the corresponding author(s).

## Supplementary Materials

The following supporting information can be downloaded at https://www.mdpi.com/article/10.3390/membranes15050147/s1. Figure S1: The schematic diagram of concentration cell. Figure S2: I-V curves of H_2_ permeability test of Zirfon. Figure S3: I-V curves of H_2_ permeability test of PPS. Figure S4: I-V curves of H_2_ permeability test of FAA. Figure S5: I-V curves of H_2_ permeability test of BILP-PE. **Table S1**: Limiting current density *j_l_* (mA·cm^−2^), permeation flux *J_H_* × 10^7^ (L·cm^−2^·s^−1^) and H_2_ permeability *P_H_* × 10^10^ (L·cm·cm^−2^·s^−1^) of Zirfon. Table S2: Limiting current density *j_l_* (mA·cm^−2^), permeation flux *J_H_* × 10^7^ (L·cm^−2^·s^−1^) and H_2_ permeability *P_H_* × 10^10^ (L·cm·cm^−2^·s^−1^) of PPS. Table S3: Limiting current density *j_l_* (mA·cm^−2^), permeation flux *J_H_* × 10^7^ (L·cm^−2^·s^−1^) and H_2_ permeability *P_H_* × 10^10^ (L·cm·cm^−2^·s^−1^) of FAA. Table S4: Limiting current density *j_l_* (mA·cm^−2^), permeation flux *J_H_* × 10^7^ (L·cm^−2^·s^−1^) and H_2_ permeability *P_H_* × 10^10^ (L·cm·cm^−2^·s^−1^) of BILP-PE. Table S5: The comparison of H_2_ permeability by different methods (×10^14^ mol·cm·s^−1^·cm^−2^·kPa^−1^). References [23,24] are cited in the Supplementary Materials.
