# Supplementary material for: In-Situ Measurement of Gas Permeability for Membranes in Water Electrolysis
Source: Membranes (Basel). 2025 May 13;15(5):147. doi: 10.3390/membranes15050147 (PMC12113548; doi:10.3390/membranes15050147)
Supplement: Supplementary file 1 [file membranes-15-00147-s001.zip › membranes-3567823-supplementary.pdf]

Supporting Information

In-situ measurement of gas permeability for membranes in water electrolysis

Shuaimin Li[1,2], Chuan Song[1,2], Li Xu[1,2,3], Yuxin Wang[1,2], Wen Zhang[1,2,3*]

1 State Key Laboratory of Chemical Engineering and Low-Carbon Technology, School of Chemical Engineering and Technology, Tianjin University, Tianjin 300350, China

2 Tianjin Key Laboratory of Membrane Science & Desalination Technology, School of Chemical Engineering and Technology, Tianjin University, Tianjin 300350, China

3 National Industry-Education Integration Platform of Energy Storage, Tianjin University, Tianjin, China 300350

*Corresponding Author Email: zhang_wen@tju.edu.cn

At 0 V, the hydrogen concentration at electrode 2 is greater than at electrode 1. At this time, the system forms a concentration cell, so a negative current is generated. Fig. S1 The schematic diagram of concentration cell.

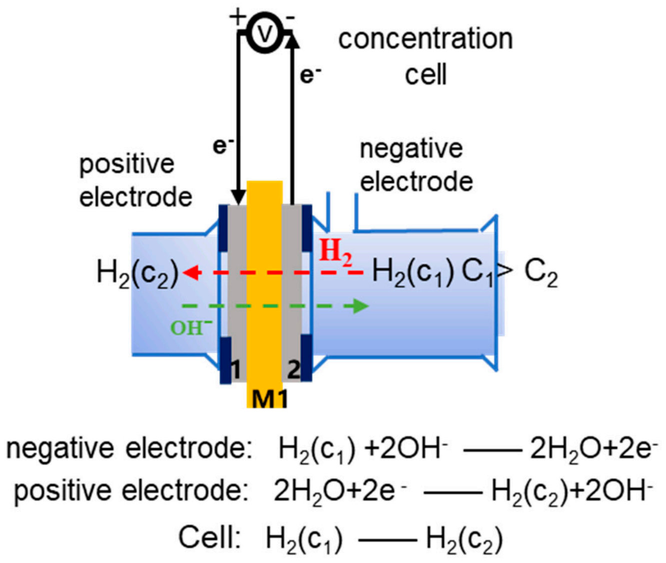

Fig. S1 The schematic diagram of concentration cell

The LSV curves (1.1~1.5 V sections) of H_2 permeability of the four membranes, Zirfon, PPS, FAA, and BILP-PE, measured by the in-situ method at different conditions, are shown in Figs. S2, S3, S4 and S5.

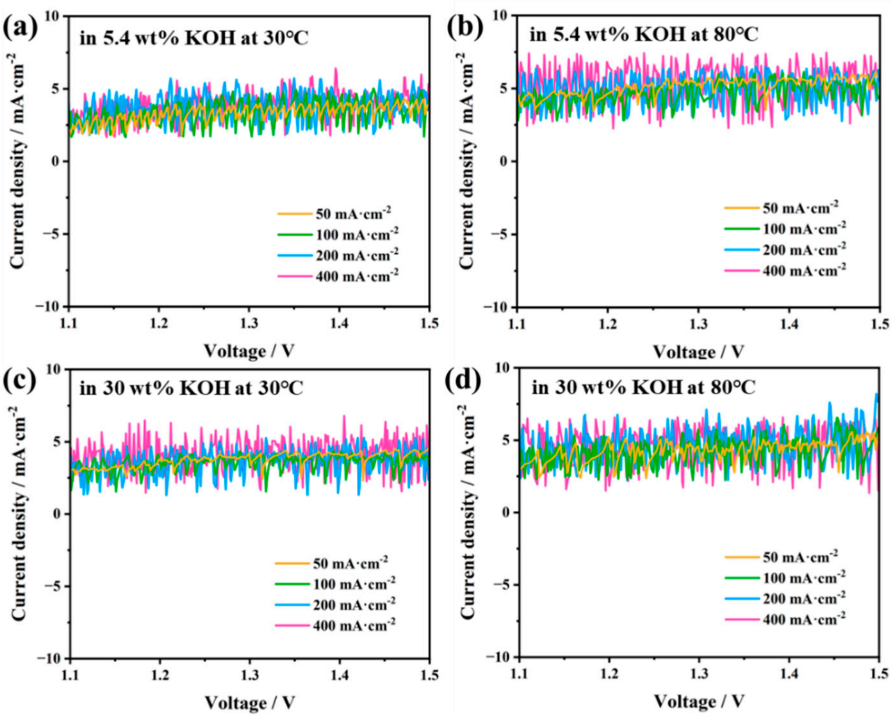

Fig. S2 I-V curves of H_2 permeability test of Zirfon

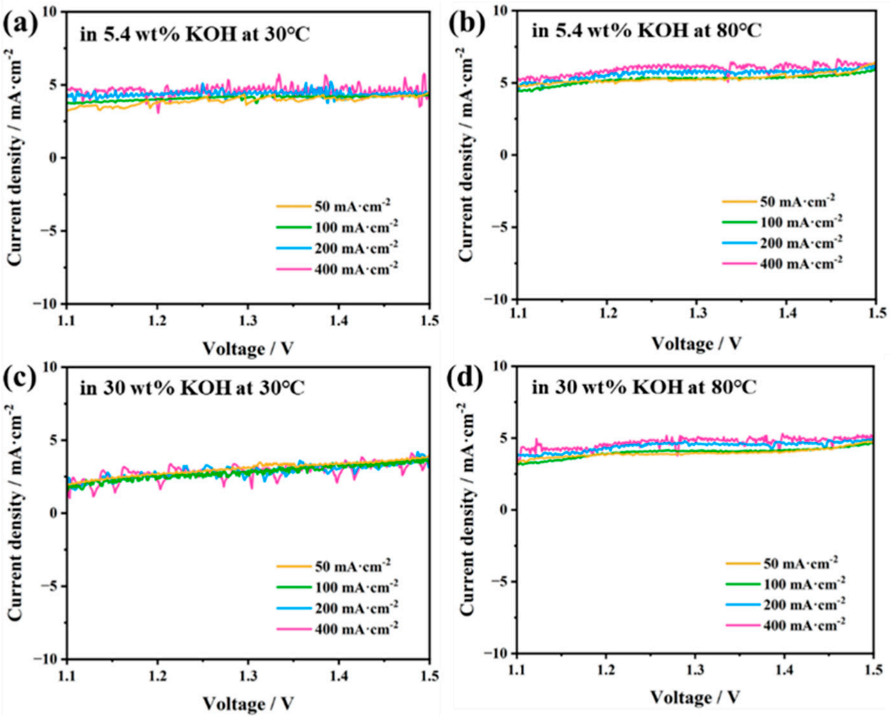

Fig. S3 I-V curves of H_2 permeability test of PPS

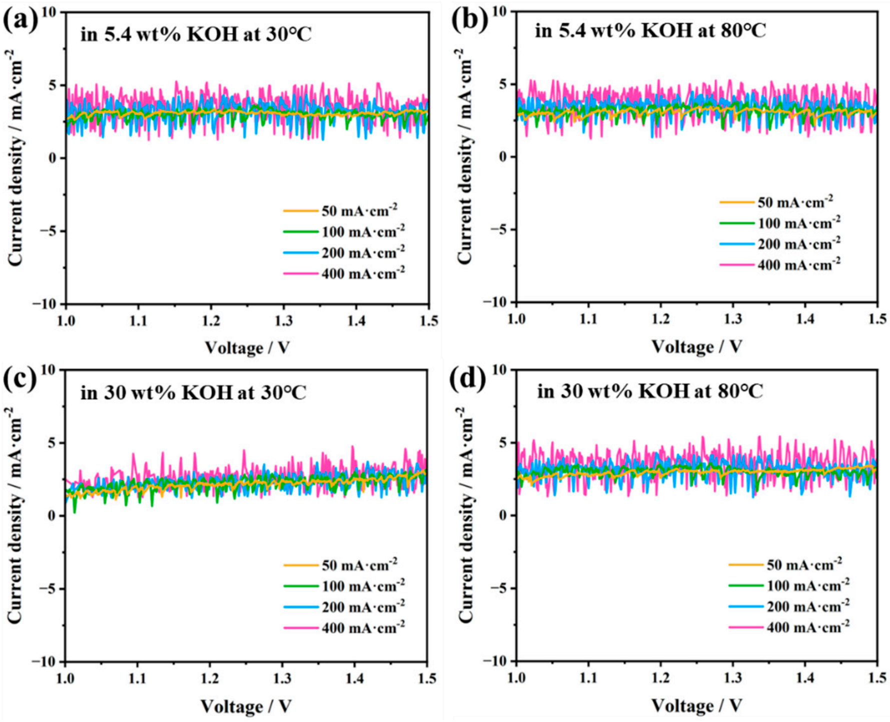

Fig. S4 I-V curves of H_2 permeability test of FAA

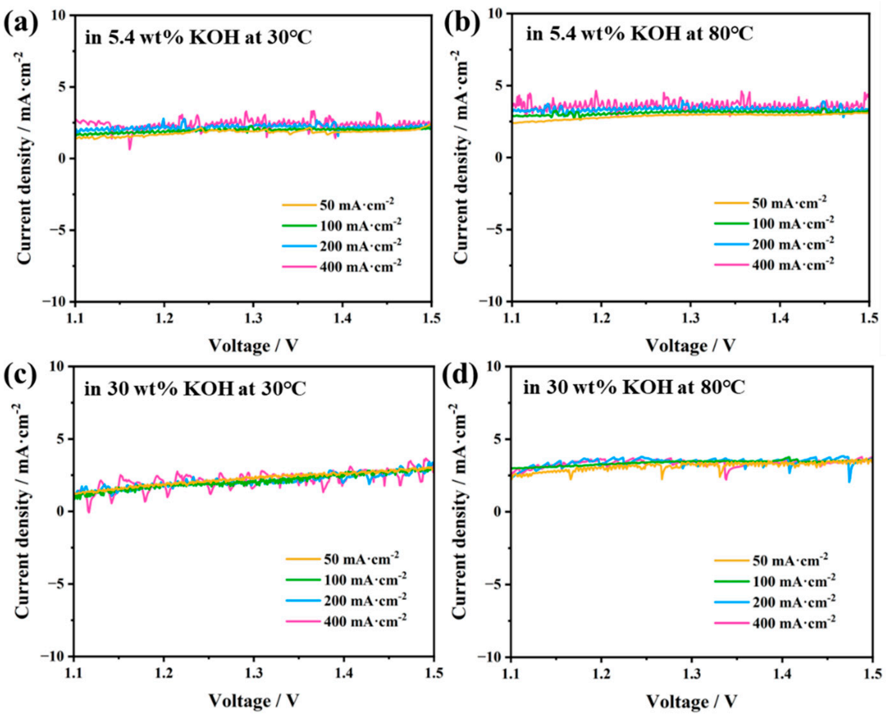

Fig. S5 I-V curves of H_2 permeability test of BILP-PE

The HOR limiting current densities jl of the membranes and the H_2 permeation fluxes J_H and H_2 permeability P_H calculated from Eqs. 1 and 2 are listed in Tables S1, S2, S3, and S4.

Table S1. Limiting current density j_l ($mA\cdot cm^{-2}$), permeation flux $J_H\times10^7$ ($L\cdot cm^{-2}\cdot s^{-1}$) and H_2 permeability $P_H\times10^{10}$ ($L\cdot cm\cdot cm^{-2}\cdot s^{-1}$) of Zirfon.

			50 $mA\cdot cm^{-2}$	100 $mA\cdot cm^{-2}$	200 $mA\cdot cm^{-2}$	400 $mA\cdot cm^{-2}$
5.4 wt% KOH	30 °C	j_l	3.42	3.55	3.58	3.61
		J_H	3.97	4.12	4.16	4.19
		P_H	199	206	208	210
	80 °C	j_l	5.11	5.14	5.19	5.49
		J_H	5.93	5.97	6.02	6.37
		P_H	297	299	301	319
30 wt% KOH	30 °C	j_l	2.67	2.74	2.83	2.91
		J_H	3.10	3.18	3.29	3.38
		P_H	155	159	165	169
	80 °C	j_l	4.01	4.11	4.39	4.53
		J_H	4.65	4.77	5.10	5.26
		P_H	233	239	255	263

Table S2. Limiting current density j_l (mA·cm^{-2}), permeation flux J_H×10^7 (L·cm^{-2}·s^{-1}) and H_2 permeability P_H×10^{10} (L·cm·cm^{-2}·s^{-1}) of PPS.

			50 mA·cm^{-2}	100 mA·cm^{-2}	200 mA·cm^{-2}	400 mA·cm^{-2}
5.4 wt% KOH	30 °C	j_l	3.75	3.81	3.84	3.88
		J_H	4.35	4.42	4.46	4.50
		P_H	296	301	303	306
	80 °C	j_l	5.15	5.33	5.49	5.88
		J_H	5.98	6.19	6.37	6.83
		P_H	407	421	433	464
30 wt% KOH	30 °C	j_l	2.99	3.02	3.07	3.15
		J_H	3.47	3.51	3.56	3.66
		P_H	236	239	242	249
	80 °C	j_l	4.16	4.31	4.53	4.65
		J_H	4.83	5.00	5.26	5.40
		P_H	328	340	358	367

Table S3. Limiting current density j_l (mA·cm^{-2}), permeation flux J_H×10^{7} (L·cm^{-2}·s^{-1}) and H_2 permeability P_H×10^{10} (L·cm·cm^{-2}·s^{-1}) of FAA.

			50 mA·cm^{-2}	100 mA·cm^{-2}	200 mA·cm^{-2}	400 mA·cm^{-2}
5.4 wt% KOH	30 °C	j_l	2.84	3.04	3.08	3.09
		J_H	3.30	3.53	3.58	3.59
		P_H	24.8	26.5	26.9	26.9
	80 °C	j_l	3.1	3.17	3.22	3.27
		J_H	3.60	3.68	3.74	3.80
		P_H	27.0	27.6	28.1	28.5
30 wt% KOH	30 °C	j_l	2.13	2.19	2.22	2.23
		J_H	2.47	2.54	2.58	2.59
		P_H	18.5	19.1	19.4	19.4
	80 °C	j_l	3.03	3.07	3.18	3.25
		J_H	3.52	3.56	3.69	3.77
		P_H	26.4	26.7	27.7	28.3

Table S4. Limiting current density j_l (mA·cm^{-2}), permeation flux J_H×10^{7} (L·cm^{-2}·s^{-1}) and H_2 permeability P_H×10^{10} (L·cm·cm^{-2}·s^{-1}) of BILP-PE.

			50 mA·cm^{-2}	100 mA·cm^{-2}	200 mA·cm^{-2}	400 mA·cm^{-2}
5.4 wt% KOH	30 °C	j_l	1.97	1.99	2.09	2.1
		J_H	2.29	2.31	2.43	2.44
		P_H	3.46	3.49	3.67	3.68
	80 °C	j_l	3.03	3.04	3.08	3.09
		J_H	3.52	3.53	3.58	3.59
		P_H	5.32	5.33	5.41	5.42
30 wt% KOH	30 °C	j_l	1.55	1.56	1.58	1.61
		J_H	1.80	1.81	1.83	1.87
		P_H	2.72	2.73	2.76	2.82
	80 °C	j_l	2.94	2.95	2.96	3.03
		J_H	3.41	3.42	3.44	3.52
		P_H	5.15	5.16	5.19	5.32

The H_2 permeability of different membranes in the in-situ method and other previous methods in the following table.

Table S5. The comparison of H_2 permeability by different methods ($\times 10^{14}$ mol·cm·s^{-1}·cm^{-2}·kPa^{-1})

Membranes	Electrochemical method		Volumetric method	Concentration method
	In-suit	Gas-phase		
PPS	125×10^{1}	423		
Zirfon	896	273		
FAA	96.3	35.8		
BILP-PE	18.1	4.26		
Nafion 117			21.4[23]	
Nafion 117				18.0*[24]

*Assuming the measurement is taken under atmospheric pressure.

References:

23. Sakai, T.; Takenaka, H.; Wakabayashi, N.; Kawami, Y.; Torikai, E. Gas Permeation Properties of Solid Polymer Electrolyte (SPE) Membranes. *Journal of The Electrochemical Society* **1985**, *132*, 1328, doi:10.1149/1.2114111.
24. Broka, K.; Ekdunge, P. Oxygen and hydrogen permeation properties and water uptake of Nafion® 117 membrane and recast film for PEM fuel cell. *Journal of Applied Electrochemistry* **1997**, *27*, 117-123, doi:10.1023/A:1018469520562.
